# Mammography radiomics features at diagnosis and progression-free survival among patients with breast cancer

**DOI:** 10.1038/s41416-022-01958-5

**Published:** 2022-09-01

**Authors:** Chuanxu Luo, Shuang Zhao, Cheng Peng, Chengshi Wang, Kejia Hu, Xiaorong Zhong, Ting Luo, Juan Huang, Donghao Lu

**Affiliations:** 1 Laboratory of Molecular Diagnosis of Cancer, Clinical Research Center for Breast, West China Hospital, Sichuan University, Chengdu, China; 2 Department of Radiology, West China Hospital of Sichuan University, Chengdu, China; 3 Channing Division of Network Medicine, Brigham and Women’s Hospital, Boston, MA USA; 4 Department of breast surgery, Sichuan Cancer Hospital and Institute, Sichuan Cancer Center, School of Medicine, University of Electronic Science and Technology of China, Chengdu, China; 5 Unit of Integrative Epidemiology, Institute of Environmental Medicine, Karolinska Institute, Stockholm, Sweden; 6 Department of Head, Neck and Mammary Gland Oncology, Cancer Center, West China Hospital, Sichuan University, Chengdu, China; 7 West China Biomedical Big Data Center, West China Hospital, Sichuan University, Chengdu, China

**Keywords:** Cancer imaging, Breast cancer

## Abstract

**Background:**

The associations between mammographic radiomics and breast cancer clinical endpoints are unclear. We aimed to identify mammographic radiomics features associated with breast cancer prognosis.

**Methods:**

Nested from a large breast cancer cohort in our institution, we conducted an extreme case-control study consisting of 207 cases with any invasive disease-free survival (iDFS) endpoint <5 years and 207 molecular subtype-matched controls with >5-year iDFS. A total of 632 radiomics features in craniocaudal (CC) and mediolateral oblique (MLO) views were extracted from pre-treatment mammography. Logistic regression was used to identify iDFS-associated features with multiple testing corrections (Benjamini–Hochberg method). In a subsample with RNA-seq data (*n* = 96), gene set enrichment analysis was employed to identify pathways associated with lead features.

**Results:**

We identified 15 iDFS-associated features from CC-view yet none from MLO-view. S(1,−1)SumAverg and WavEnLL_s-6 were the lead ones and associated with favourable (OR 0.64, 95% CI 0.42–0.87, *P* = 0.01) and poor iDFS (OR 1.53, 95% CI 1.31–1.76, *P* = 0.01), respectively. Both features were associated with eight pathways (primarily involving cell cycle regulation) in tumour but not adjacent normal tissues.

**Conclusion:**

Our findings suggest mammographic radiomics features are associated with breast cancer iDFS, potentially through pathways involving cell cycle regulation.

## Introduction

Breast cancer is the most commonly diagnosed cancer and the leading cause of cancer deaths in women [1]. Although targeted therapy has significantly improved breast cancer survival [2], breast cancer mortality remains the fifth leading cause of cancer mortality worldwide, with 685,000 deaths in 2020 [1]. In addition to estrogen receptor (ER), progesterone receptor (PR) and human epidermal growth factor receptor 2 status (HER2), best-validated gene expression assays (e.g. Oncotype Dx [3], MammaPrint [4] and PAM50 [5]) may assist clinical decision-making to improve prognosis. However, non-invasive and low-cost markers, independent of tumour characteristics, in the prediction of breast cancer prognosis are still lacking.

In the past decade, advances in medical image analysis promoted the process of radiomics. Based on the quantitative features of intensity, shape, size or volume and texture, radiomics convert images into higher dimensional data for improved decision support [6]. Radiomics in breast cancer include breast magnetic resonance imaging (MRI), mammography and ultrasonography [7]. Mounting evidence demonstrated the associations of MRI features with diagnosis [8], prognosis [9], molecular subtyping [10] and response to neoadjuvant chemotherapy in breast cancer patients [7, 11]. For instance, pre-operative MRI radiomics signatures were proved to predict invasive disease-free survival (iDFS) in patients with invasive breast cancer [12, 13].

Mammography is a widely applied approach for breast cancer screening and diagnosis. Several automated feature extraction methods have been developed for mammography radiomics features, such as radical edge-gradient analysis [14], deep CNN model [15] and radiomics software (e.g. MAZDA [16], A.K. software [17] and TIK-SNAP software [18]). Although manual mass segmentation is needed for some methods before processing, a favourable agreement has been shown by different readers [19]. It has been shown that mammographic radiomics features were predictive for mass classification [17, 19] and level of tumour-infiltrating lymphocytes [18, 19] and were associated with oncotype DX test recurrence score [20]. However, it is unclear whether mammographic radiomics features are associated with breast cancer clinical endpoints (e.g. iDFS or overall survival).

In the present study, we aimed to identify mammographic radiomics features associated with breast cancer prognosis and explore the potential biological basis using RNA-seq data.

## Methods

### Study design

The Breast Cancer Cohort from our institution is a prospective cohort of patients who were diagnosed with breast cancer in our institution from January 2009 onwards [20]. Information on tumour characteristics, such as tumour size (the largest tumour diameter), tumour stage (TNM stage according to the AJCC Cancer Staging Manual), histologic grade (Scarff-Bloom-Richardson histologic grading system [21]) and molecular subtype [22], as well as treatment, was collected from medical records [23]. Patients were followed every 4 months in the first 3 years after surgery, every 6–12 months in the fourth and fifth years and annually thereafter. Follow-up was operated by office visit, telephone or postal contact. This study was approved by the Clinical Test and Biomedical Ethics Committee of our institution (No. 2019-16). Informed consent was obtained from all patients.

**Fig. 1:**
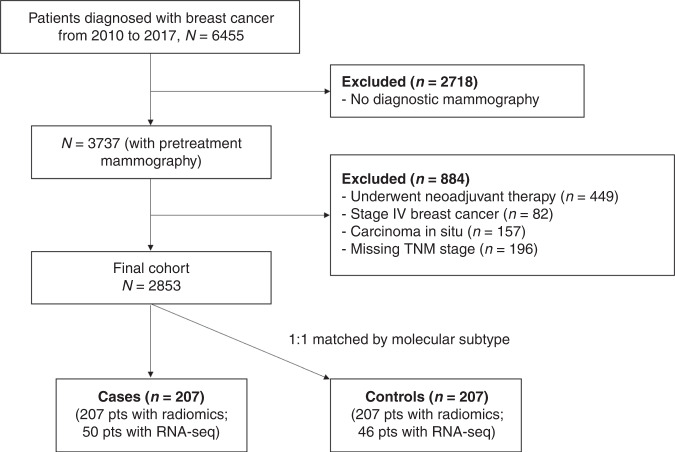
Flow diagram of patient selection. The flowchart depicts the patient selection process from a prospective cohort of patients being diagnosed with breast cancer in our institution between January 2010 and February 2017.

Both diagnostic ultrasound and mammography were routinely performed for patients with suspicious breast mass at our institution. The digital database of mammography images was available from January 2010. Between January 2010 and February 2017, 6455 patients were enrolled on the cohort; among them, 3737 (57.9%) patients received mammography in our institution before surgery. Patients were further excluded according to the following criteria: underwent neoadjuvant therapy (*n* = 449); with distant metastases (*n* = 82); carcinoma in situ (*n* = 157) or with missing information on tumour stage, i.e. unknown number of positive lymph nodes (*n* = 8) and tumour size (*n* = 188). Subsequently, 2853 patients with pre-surgical mammography were eligible for this study (Fig. 1).

To optimise the cost-effectiveness and maximise the statistical power, we performed a matched extreme case-control design (comparing patients with the poorest prognosis with those with the most favourable prognosis) which has been proved to successfully identify prognostic biomarkers associated with cancer prognosis [24]. During the follow-up from diagnosis through June 2020 (median: 5.2 years), 207 patients developed iDFS endpoints [25] (including any local or regional recurrence, distant metastasis, contralateral breast cancer, secondary primary cancer, cancer-specific death and death from other causes) during the first 5 years after cancer diagnosis (cases). Patients without any clinical events of iDFS who survived at least 5 years after cancer diagnosis were considered controls (*n* = 1451). Controls were randomly selected and 1:1 matched on molecular subtype [22]. Finally, 414 patients (207 cases) were included for analysis; fresh-frozen breast tumour tissues were available for 50 cases and 46 controls.

### Mammography radiomics features

Both diagnostic ultrasound and mammography were routinely performed for patients with suspicious breast mass for purpose of diagnosing/staging at our institution, a tertiary medical centre. Because no formal mammography screening program has been established in our province, most breast cancers were detected due to symptoms or breast self-examination.

All mammogram images were obtained from the GE SenoBright full-field digital mammography systems (GE Healthcare, Chicago, IL). Leveraging the free radiomics platform MAZDA software (Technical University of Lodz) [16], an experienced and specialised radiologist (one of the first authors having subspecialty experience in breast radiology) manually outlined tumour contours in craniocaudal (CC) and mediolateral oblique (MLO) views of each patient and extracted radiomics features of the lesion area. MAZDA computed six categories of features: histogram, gradient, co-occurrence matrix, run-length matrix (RLM), autoregressive model and Haar wavelet groups. A total of 316 mammography radiomics features were extracted from each view. The values of each feature were normalised through z-score transformation.

### RNA sequencing

Fresh-frozen breast tumour tissues and paired adjacent normal tissues were donated at the surgery. RNA integrity was examined using the RNA Nano 6000 Assay Kit of the Agilent Bioanalyzer 2100 system and RNA Integrity Number (RIN) > 7.0 was required to pass the quality control. RNA sequencing was performed on the Illumina Hi-Seq, Paired-end 150. Raw RNA-seq data were processed according to nfcore/rnaseq: a bioinformatics analysis pipeline for RNA sequencing data (https://nf-co.re/rnaseq/1.0/). Fragments Per Kilobase Million (FPKM) of each gene were calculated by dividing the read count by the gene length and the total number of reads mapped to protein-coding genes. A total of 19,448 genes were profiled. The FPKM values were further normalised using z-score transformation.

### Statistical analysis

Clinical characteristics, including age at diagnosis, menopausal status, molecular subtype, tumour stage, histologic grade, hormone therapy, chemotherapy and radiotherapy, were compared between patients with poor iDFS (cases) and those with favourable iDFS (controls) using *t*-test for continuous variables or Chi-square test for categorical ones. Using logistic regression, odds ratios (ORs) and 95% confidence intervals (CIs) of iDFS endpoints were estimated for mammography radiomics features from CC and MLO views, respectively. As described above, iDFS endpoints included any local or regional recurrence, distant metastasis, contralateral breast cancer, secondary primary cancer, cancer-specific death and death from other causes. Multiple testing was corrected using Benjamini–Hochberg method to control for the false discovery rate (FDR). We presented ORs without adjustment (Model 1), ORs adjusted for demographic characteristics (age at diagnosis and menopausal status; Model 2) and ORs additionally adjusted for tumour characteristics (molecular subtype, tumour stage and histologic grade; Model 3). Radiomics features with significant associations in Model 3 were considered as lead markers. In a sensitivity analysis, we further adjusted for treatment modes (receipt of chemotherapy, hormone therapy and radiotherapy; Model 4), which should be considered as potential mediators rather than confounders. Many radiomics features were highlighted as correlated. To reduce multiple testing, we used a correlation matrix to examine clusters and identify top independent markers for subsequent analyses, in which Model 3 was applied.

Leveraging the RNA-seq data from breast cancer tissues and paired adjacent normal tissues, we examined the associations between top independent markers and the expression levels of individual genes using linear regression and visualised them in volcano plots. *P* values were corrected using FDR. To shed light on the biological mechanisms, we performed gene set enrichment analysis using a camera package and identified hallmark and KEGG pathways associated with top independent markers. The gene lists of candidate pathways were downloaded from the Gene Set Enrichment Analysis (http://www.gsea-msigdb.org/gsea/index.jsp).

All statistical tests were two-tailed and conducted using R version 3.5.0 (R Foundation for Statistical Computing, Vienna, Austria).

## Results

Compared to patients with favourable iDFS (controls; *n* = 207), patients with poor iDFS (cases; *n* = 207) were more likely to be post-menopausal at diagnosis (43.0% vs. 32.4%, *P* = 0.033), to have advanced tumour stage (42.0% vs. 15.0%, *P* < 0.001) and high histologic grade (65.7% vs. 54.6%, *P* = 0.027) and to receive no hormone therapy (57.0% vs. 69.1%, *P* = 0.015; Table 1). Similar patterns were observed for patients whose tumours have been sequenced for RNA, although the tumour stage was significantly different between the two groups (50.0% vs. 10.9%, *P* < 0.001; Supplementary Table S1).

**Table 1:** Clinical characteristics of breast cancer patients with poor (cases) and favourable invasive disease-free survival (controls): an extreme case-control study.

		Controls	Cases	*P*
Number		207	207	
		Mean (SD)	Mean (SD)	
Age, years		47.74 (8.69)	49.14 (10.58)	0.140
		*N* (%)	*N* (%)	
Menopausal status	No	140 (67.6)	118 (57.0)	0.033
	Yes	67 (32.4)	89 (43.0)	
Molecular subtype	Luminal A	12 (5.8)	12 (5.8)	0.999
	Luminal B	119 (57.5)	119 (57.5)	
	HER2 positive	29 (14.0)	29 (14.0)	
	TNBC	32 (15.5)	32 (15.5)	
	Indeterminate	15 (7.2)	15 (7.2)	
Tumour stage	I	58 (28.0)	22 (10.6)	<0.001
	II	118 (57.0)	98 (47.3)	
	III	31 (15.0)	87 (42.0)	
Histologic grade	I–II	94 (45.4)	71 (34.3)	0.027
	III	113 (54.6)	136 (65.7)	
Hormone therapy	No	64 (30.9)	89 (43.0)	0.015
	Yes	143 (69.1)	118 (57.0)	
Chemotherapy	No	6 (2.9)	11 (5.3)	0.322
	Yes	201 (97.1)	196 (94.7)	
Radiotherapy	No	131 (63.3)	133 (64.3)	0.919
	Yes	76 (36.7)	74 (35.7)	

*N* number, *SD* standard deviation, *HER2* human epidermal growth factor receptor 2, *TNBC* triple-negative breast cancer.

### Identifying features associated with iDFS

In our primary model, where we adjusted for demographic and tumour characteristics, 15 out of 316 mammography radiomics features from CC-view were significantly associated with iDFS after multiple testing corrections (all *P* < 0.05; Table 2, Model 3). These associations remained significant with additional adjustments for treatment modes (Supplementary Table S2). In a sensitivity analysis, we excluded cases who died of non-breast-cancer causes and yielded similar findings (Supplementary Table S3). Other CC-view features were not associated with iDFS (Supplementary Table S4). Moreover, no features from the MLO-view were associated with iDFS after multiple testing correlations (all *P* > 0.05; Supplementary Table S5). Although none of MLO-view features was identified as lead features, three MLO-view features [GrKurtosis, Skewness and S(1,0)SumAverg] appeared associated with iDFS (empirical *P* < 0.05; Supplementary Table S6).

**Table 2:** Associations of lead mammography radiomics features at diagnosis with invasive disease-free survival among patients with breast cancer^a^, after multiple testing correlation.

Features	Mean (SD)		Model 1^b^		Model 2^c^		Model 3^d^	
	Controls	Cases	OR (95% CI)	*P*	OR (95% CI)	*P*	OR (95% CI)	*P*
S(1,-1)SumAverg	0.13 (0.95)	−0.13 (1.03)	0.76 (0.56–0.96)	0.043	0.71 (0.50–0.93)	0.035	0.64 (0.42–0.87)	0.010
S(2,0)SumAverg	0.14 (0.97)	−0.14 (1.01)	0.76 (0.55–0.96)	0.043	0.72 (0.51–0.92)	0.035	0.66 (0.44–0.88)	0.010
S(2,−2)SumAverg	0.16 (1.00)	−0.16 (0.98)	0.72 (0.52–0.93)	0.043	0.68 (0.47–0.89)	0.016	0.64 (0.41–0.87)	0.010
S(3,0)SumAverg	0.14 (1.00)	−0.14 (0.98)	0.74 (0.54–0.94)	0.043	0.70 (0.49–0.91)	0.023	0.66 (0.44–0.88)	0.010
S(3,−3)SumAverg	0.16 (1.01)	−0.16 (0.97)	0.72 (0.52–0.92)	0.043	0.68 (0.47–0.89)	0.014	0.65 (0.42–0.87)	0.010
S(4,−4)SumAverg	0.16 (1.01)	−0.16 (0.96)	0.72 (0.51–0.92)	0.043	0.68 (0.47–0.89)	0.014	0.65 (0.43–0.88)	0.010
S(5,−5)SumAverg	0.16 (1.02)	−0.16 (0.96)	0.72 (0.52–0.92)	0.043	0.68 (0.47–0.89)	0.014	0.66 (0.43–0.88)	0.010
WavEnLL_s-6	−0.19 (1.05)	0.19 (0.91)	1.49 (1.28–1.69)	0.026	1.56 (1.35–1.77)	0.011	1.53 (1.31–1.76)	0.010
S(4,0)SumAverg	0.15 (1.01)	−0.15 (0.97)	0.74 (0.54–0.94)	0.043	0.70 (0.49–0.91)	0.020	0.67 (0.44–0.89)	0.011
S(1,0)SumAverg	0.11 (0.92)	−0.11 (1.07)	0.80 (0.60–1.00)	0.062	0.76 (0.55–0.96)	0.066	0.67 (0.45–0.89)	0.012
S(5,0)SumAverg	0.15 (1.02)	−0.15 (0.96)	0.74 (0.54–0.94)	0.043	0.70 (0.49–0.91)	0.020	0.67 (0.45–0.89)	0.012
WavEnLL_s-7	−0.15 (1.04)	0.15 (0.94)	1.36 (1.16–1.55)	0.043	1.43 (1.22–1.63)	0.020	1.48 (1.25–1.70)	0.017
WavEnLL_s-5	−0.18 (1.05)	0.18 (0.92)	1.45 (1.25–1.66)	0.026	1.51 (1.30–1.72)	0.011	1.48 (1.25–1.70)	0.018
WavEnHL_s-5	0.19 (1.02)	−0.19 (0.95)	0.66 (0.43–0.88)	0.026	0.64 (0.41–0.87)	0.011	0.69 (0.46–0.92)	0.028
WavEnLL_s-4	−0.18 (1.05)	0.18 (0.91)	1.46 (1.25–1.66)	0.026	1.50 (1.29–1.71)	0.011	1.44 (1.22–1.67)	0.031

*SD* standard deviation, *OR* odds ratio, *CI* confidence interval.

^a^Only features with FDR-corrected *P-*values less than 0.05 in Model 3 were present.

^b^Crude model.

^c^Estimates were adjusted for age and menopausal status.

^d^Estimates were adjusted for age, menopausal status, molecular subtype, tumour stage and histologic grade.

Using the correlation matrix analysis, we identified two clusters from the 15 lead features from CC-view (Supplementary Fig. S1). S(1,−1)SumAverg and WavEnLL_s-6 were the top features (with strongest association with iDFS) in each cluster, and associated with favourable iDFS (OR, 0.64; 95% CI, 0.42–0.87; *P* = 0.010; Table 2) and poor iDFS (OR, 1.53; 95% CI, 1.31–1.76; *P* = 0.010; Table 2), respectively. Feature S(1,−1)SumAverg belongs to the category of co-occurrence matrix, while feature WavEnLL_s-6 is part of the Haar wavelet category. In stratified analyses, stronger associations were found in pre-menopausal patients for both features and patients with Luminal A tumour for feature S(1,−1)SumAverg (P-for-interaction < 0.05; Supplementary Table S7). Similar associations were noted for both features across tumour stage and histologic grade groups.

### Exploring the biological basis for identified features

Feature WavEnLL_s-6 was significantly associated with a more advanced tumour stage (*P* = 0.029; Supplementary Table S8) but not associated with molecular subtype or histologic grade. Feature S(1,−1)SumAverg was not associated with tumour characteristics.

**Fig. 2:**
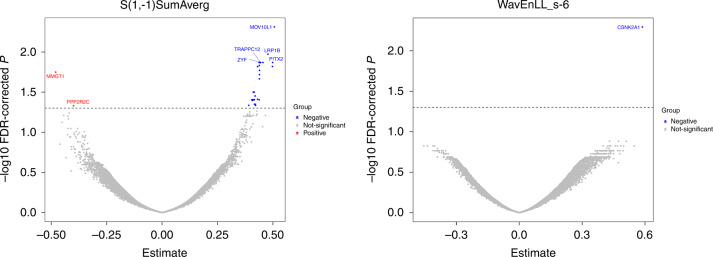
Gene expression signatures associated with top independent mammography radiomics features at diagnosis among patients with breast cancer. Red-coloured genes had negative estimates, while blue-coloured genes had positive estimates. Estimates were adjusted for age, menopausal status, molecular subtype, tumour stage and histologic grade. FDR false discovery rate.

In a subsample of patients whose tumours were profiled for RNA (*n* = 96), feature S(1,−1)SumAverg was significantly associated with the expression of gene *MOV10L1*, *LRP1B*, *ZFY*, *PITX2*, *TRAPPC12*, *MMGT1* and *PPP2R2C* (all *P* < 0.05; Fig. 2 and Supplementary Table S9). Feature WavEnLL_s-6 was positively associated with the expression of gene *CSNK2A1* (*P* = 0.005; Fig. 2 and Supplementary Table S9). The above associations were not observed in paired adjacent normal tissues (*n* = 60; Supplementary Table S9).

In pathway analysis, we further found that feature S(1,−1)SumAverg was significantly associated with upregulated signalling of 8 pathways, including G2M checkpoint pathway, *E2F*_target genes, *MYC* targets v1 and v2 (two subgroups of genes regulated by *MYC*—version 1 and—version 2), DNA replication, mismatch repair pathway, cell cycle pathway and the ubiquitin-proteasome pathway (all *P* < 0.05; Table 3). The majority of these pathways were essential for DNA replication, cell cycle progression and DNA damage repair. Feature WavEnLL_s-6 was similarly associated with the aforementioned pathways and with downregulated signalling of the ribosome pathway (all *P* < 0.05; Table 3). However, in paired adjacent normal tissues (*n* = 60), both features were not associated with the aforementioned pathways except *MYC* targets v2 in an opposite direction for feature WavEnLL_s-6 (Table 3).

**Table 3:** Pathways associated with top independent mammography radiomics features at diagnosis among patients with breast cancer using gene set enrichment analysis^a^, in tumour tissues and adjacent normal tissues.

				Tumour		Normal	
			*N* of Genes	Direction	P*	Direction	P*
S(1,−1)SumAverg	Hallmark	G2M_CHECKPOINT	193	Up	4.99E-07	Down	0.751
		E2F_TARGETS	198	Up	4.99E-07	Down	0.751
		MYC_TARGETS_V1	195	Up	1.98E-04	Up	0.935
		MYC_TARGETS_V2	58	Up	9.25E-05	Down	0.107
	KEGG	DNA_REPLICATION	36	Up	8.83E-05	Down	0.947
		MISMATCH_REPAIR	23	Up	0.023	Down	0.947
		CELL_CYCLE	124	Up	0.043	Up	0.994
		PROTEASOME	44	Up	0.043	Up	0.366
WavEnLL_s-6	Hallmark	G2M_CHECKPOINT	193	Up	2.81E-07	Down	0.588
		E2F_TARGETS	198	Up	2.80E-07	Down	0.582
		MYC_TARGETS_V1	195	Up	1.16E-04	Down	0.749
		MYC_TARGETS_V2	58	Up	7.26E-05	Down	0.016
	KEGG	DNA_REPLICATION	36	Up	2.54E-05	Down	0.889
		MISMATCH_REPAIR	23	Up	0.011	Down	0.909
		RIBOSOME	86	Down	0.011	Up	0.684
		CELL_CYCLE	124	Up	0.031	Down	0.970
		PROTEASOME	44	Up	0.041	Up	0.684

*N* number.

*FDR-corrected *P-*value.

^a^This analysis was performed in breast cancer patients whose tumours have been sequenced for RNA (*n* = 96) and adjusted for age, menopausal status, molecular subtype, tumour stage and histologic grade.

## Discussion

To the best of our knowledge, this is the first study to identify mammography radiomics features associated with invasive disease-free survival among patients with invasive breast cancer. In the extreme case-control study nested from a large prospective cohort, two independent features from CC-view, S(1,−1)SumAverg and WavEnLL_s-6, were associated with invasive disease-free survival, independent of clinical characteristics and after stringent correction for multiple comparisons. Moreover, both features were associated with gene signatures of pathways responsible for DNA replication, cell cycle progression and DNA damage repair in tumour tissue, but not in paired adjacent normal tissue. These findings shed light on the potential biological mechanisms linking identified features to breast cancer prognosis. Although these markers are to be confirmed in an independent setting, the highlighted biological processes underlying these features strongly refute the possibility of findings by pure chance.

In a previous study of 350 patients with ER-positive breast cancer, Woodard et al. found that mammographic breast density, calcification morphology and mass margins were correlated with a prognostic biomarker—the Oncotype DX recurrence score [20]. However, these features were derived from the BI-RADS, which is subjective to the observer’s visual interpretation [26]; and the associations were not confirmed using clinical endpoints. By contrast, mathematical extraction of mammography radiomics features is more objective and feasible for application. In the present study, we also illustrated that the identified radiomics features were associated with iDFS endpoints; and the associations were robust across molecular subtypes and independent of clinical characteristics.

One of the two top independent features, S(1,−1)SumAverg, is under the category of co-occurrence matrix [27]. Using the RNA-seq data, we showed that feature S(1,−1)SumAverg was associated with expression levels of genes (i.e. *LRP1B* and *PITX2)* in tumour tissue but not in adjacent normal tissue. Both *LRP1B* [28] and *PITX2* [29] are involved in the regulation of the cell cycle and DNA damage repair. In the pathway analysis, we further illustrated that feature S(1,−1)SumAverg was primarily associated with the signalling of pathways playing a pivotal role in DNA replication, cell cycle progression and DNA damage repair, specifically in tumour tissue. These pathways are critical for tumour phenotypes and patient survival [30, 31]. Taken together, our findings lend support to that feature S(1,−1)SumAverg may capture the underlying biological process of cell cycle regulation in breast tumours and therefore is linked to the prognosis.

The other feature, WavEnLL_s-6, is under the category of Haar wavelet [16, 32]. Our data showed that feature WavEnLL_s-6 was associated with *CSNK2A1*expression, specifically in tumour tissue. It is known that *CSNK2A1* promotes proliferation and invasiveness of breast cancer cells through mediating phosphorylation of SIRT6 [33]. Moreover, WavEnLL_s-6 was associated with the same pathways shown for S(1,−1)SumAverg and ribosome pathway. Ribosomal protein RPL15 upregulates the expression of cell cycle mediators and facilitates breast cancer metastasis in vivo [34]. Collectively, we elucidated that the feature WavEnLL_s-6 is associated with breast cancer prognosis potential through the biological process of DNA replication, cell cycle progression and DNA damage repair.

In contrast, we only found three MLO-view features that were empirically suggested for breast cancer iDFS. The reason for fewer hits in MLO-view is unclear. Gupta et al [35]. explored the correlations between CC-view and MLO-view Haralick texture features of breast lesions in 1350 patients. Among 13 feature clusters, they found 4 clusters, including sum average*,* had weak two-view correlation. These findings suggest that breast lesions displayed distinct textures in terms of the structure regularity in CC and MLO views. Of note, sum average cluster is one of the top features in CC-view associated with breast cancer iDFS. The low correlation between CC-view and MLO-view for this feature cluster may account for the fewer findings from the MLO-view.

One major strength of our study is the rich information on multi-omics data, which allowed us to explore the biological basis linking radiomics features to breast cancer prognosis. The present study, however, has several limitations. First, in our cohort, only 58% of patients had data on mammography prior to surgery, resulting in a limited sample size for this study. However, the clinical characteristics were largely comparable between patients with and without mammography, except that patients with mammography were slightly younger and had less advanced tumour stage (Supplementary Table S10). However, our estimates have been carefully adjusted for age and tumour stage. Moreover, in the stratified analysis, similar associations between the top features and iDFS were found across tumour stages. Stronger results were noted among pre-menopausal patients (i.e. younger ones), suggesting that the associations would slightly attenuate if including patients without mammography who had 7% more pre-menopausal women. Moreover, only patients with fresh-frozen samples were profiled for RNA and were included in the analyses of gene expression and pathway. Yet, the clinical characteristics of those with RNA-seq data were compared with those without, except that they were somewhat younger at diagnosis (Supplementary Table S11). Second, we only analysed the radiomics features within 2 months before surgery. Radiomics features may change along the disease course. Whether the temporal changes associated with prognosis warrants future research. Third, although the MAZDA platform provides objective measures on feature extraction, manual outlining of tumour areas in mammography may be subjective to the readers. However, a fairly good agreement has been shown for data extracted by different readers [19]. Fourth, we considered all-cause mortality as one of the iDFS endpoints, and patients who died of non-breast-cancer causes may not have experienced cancer progression. However, we have yielded very similar associations by excluding patients who died of non-breast-cancer causes. Fifth, it has been well-demonstrated that mammographic density predicts breast cancer risk [36]. Future research is needed to explore the association between mammographic density and breast cancer prognosis.

In conclusion, our findings suggest that mammography radiomics features S(1,−1)SumAverg and WavEnLL_s-6 from CC-view are associated with invasive disease-free survival among patients with breast cancer, potential through pathways involving DNA replication and damage response and cell cycle regulation. If confirmed in other studies, our findings may provide important evidence to incorporate top mammography radiomics features into the conventional breast cancer prognostic nomograms. These radiomics features could be easily obtained from the standard of care at minimal cost and aid healthcare providers to identify high-risk patients and navigate treatment decisions.

## Supplementary information

Supplementary Table S1

Supplementary Table S2

Supplementary Table S3

Supplementary_Table_S4

Supplementary_Table_S5

Supplementary Table S6

Supplementary Table S7

Supplementary Table S8

Supplementary Table S9

Supplementary Table S10

Supplementary Table S11

Supplementary FigureS1

Figure legends of Supplementary Figure S1

STROBE_checklist_case-control

## Data Availability

The datasets generated and/or analysed during the current study are available from the corresponding author on reasonable request.
