# Supplementary material for: Mammography radiomics features at diagnosis and progression-free survival among patients with breast cancer
Source: Br J Cancer. 2022 Sep 1;127(10):1886–92. doi: 10.1038/s41416-022-01958-5 (PMC9643418; doi:10.1038/s41416-022-01958-5)
Supplement: Supplementary file 1 — Supplementary Table S1 [file 41416_2022_1958_MOESM1_ESM.docx]

**Supplementary Table S1.** Clinical characteristics of breast cancer patients with poor (cases) and favorable invasive disease-free survival (controls) whose tumors have been sequenced for RNA.

|  |  | **Controls** | **Cases** | **P** |
| --- | --- | --- | --- | --- |
| Number |  | 46 | 50 |  |
|  |  | mean (SD) | mean (SD) |  |
| Age, years |  | 50.37 (9.20) | 50.24 (11.15) | 0.951 |
|  |  | N (%) | N (%) |  |
| Menopausal status | No | 25 (54.3) | 23 (46.0) | 0.540 |
|  | Yes | 21 (45.7) | 27 (54.0) |  |
| Molecular subtype | Luminal A | 2 (4.3) | 4 (8.0) | 0.111 |
|  | Luminal B | 22 (47.8) | 30 (60.0) |  |
|  | HER2 positive | 10 (21.7) | 7 (14.0) |  |
|  | TNBC | 7 (15.2) | 9 (18.0) |  |
|  | Indeterminate | 5 (10.9) | 0 (0.0) |  |
| Tumor stage | Ⅰ | 12 (26.1) | 3 (6.0) | <0.001 |
|  | Ⅱ | 29 (63.0) | 22 (44.0) |  |
|  | Ⅲ | 5 (10.9) | 25 (50.0) |  |
| Histologic grade | Ⅰ-Ⅱ | 20 (43.5) | 16 (32.0) | 0.342 |
|  | Ⅲ | 26 (56.5) | 34 (68.0) |  |
| Hormone therapy | No | 18 (39.1) | 22 (44.0) | 0.782 |
|  | Yes | 28 (60.9) | 28 (56.0) |  |
| Chemotherapy | No | 1 (2.2) | 1 (2.0) | 1.000 |
|  | Yes | 45 (97.8) | 49 (98.0) |  |
| Radiotherapy | No | 31 (67.4) | 28 (56.0) | 0.349 |
|  | Yes | 15 (32.6) | 22 (44.0) |  |

Abbreviations: N, number; SD, standard deviation; HER2, human epidermal growth factor receptor 2; TNBC, triple-negative breast cancer.
