# Supplementary material for: Mammography radiomics features at diagnosis and progression-free survival among patients with breast cancer
Source: Br J Cancer. 2022 Sep 1;127(10):1886–92. doi: 10.1038/s41416-022-01958-5 (PMC9643418; doi:10.1038/s41416-022-01958-5)
Supplement: Supplementary file 2 — Supplementary Table S2 [file 41416_2022_1958_MOESM2_ESM.docx]

**Supplementary Table S2**. Associations of lead mammography radiomics features at diagnosis with invasive disease-free survival among patients with breast cancer, additionally adjusted for cancer treatment^*^, after multiple testing correlation.

| **Features** | **Model 4^d^** | |
| --- | --- | --- |
|  | **OR (95%CI)** | **P** |
| S(1,-1)SumAverg | 0.63 (0.40-0.86) | 0.008 |
| S(2,0)SumAverg | 0.64 (0.41-0.87) | 0.008 |
| S(2,-2)SumAverg | 0.63 (0.40-0.86) | 0.008 |
| S(3,0)SumAverg | 0.64 (0.41-0.87) | 0.008 |
| S(3,-3)SumAverg | 0.64 (0.41-0.87) | 0.008 |
| S(4,-4)SumAverg | 0.65 (0.42-0.88) | 0.008 |
| S(5,-5)SumAverg | 0.65 (0.42-0.88) | 0.009 |
| WavEnLL_s-6 | 1.58 (1.34-1.81) | 0.008 |
| S(4,0)SumAverg | 0.65 (0.42-0.88) | 0.008 |
| S(1,0)SumAverg | 0.65 (0.42-0.88) | 0.008 |
| S(5,0)SumAverg | 0.66 (0.43-0.89) | 0.009 |
| WavEnLL_s-7 | 1.52 (1.28-1.75) | 0.014 |
| WavEnLL_s-5 | 1.51 (1.27-1.74) | 0.016 |
| WavEnHL_s-5 | 0.67 (0.43-0.90) | 0.016 |
| WavEnLL_s-4 | 1.47 (1.23-1.70) | 0.027 |

^*^ Features with FDR-corrected P values < 0.05 in Model 3 (Table 2) were presented.

^d^ Estimates were adjusted for age, menopausal status, molecular subtype, tumor stage, histologic grade, hormone therapy, chemotherapy and radiotherapy.

Abbreviations: OR, odds ratio; CI, confidence interval.
