# Supplementary material for: Mammography radiomics features at diagnosis and progression-free survival among patients with breast cancer
Source: Br J Cancer. 2022 Sep 1;127(10):1886–92. doi: 10.1038/s41416-022-01958-5 (PMC9643418; doi:10.1038/s41416-022-01958-5)
Supplement: Supplementary file 3 — Supplementary Table S3 [file 41416_2022_1958_MOESM3_ESM.docx]

**Supplementary Table S3.** Associations of lead mammography radiomics features at diagnosis with invasive disease-free survival among patients with breast cancer^*^, after excluding cases who died from other causes.

| **Features** | **mean (SD)** | | **Model 1^a^** | | **Model 2^b^** | | **Model 3^c^** | |
| --- | --- | --- | --- | --- | --- | --- | --- | --- |
|  | **Controls** | **Cases** | **OR (95%CI)** | **P** | **OR (95%CI)** | **P** | **OR (95%CI)** | **P** |
| WavEnLL_s-6 | -0.19 (1.05) | 0.20 (0.92) | 1.51 (1.22-1.86) | 0.035 | 1.56 (1.26-1.94) | 0.013 | 1.53 (1.22-1.92) | 0.012 |
| S(3,-3)SumAverg | 0.16 (1.01) | -0.17 (0.97) | 0.71 (0.58-0.87) | 0.041 | 0.68 (0.55-0.84) | 0.015 | 0.65 (0.52-0.81) | 0.012 |
| S(4,-4)SumAverg | 0.16 (1.01) | -0.17 (0.96) | 0.71 (0.58-0.87) | 0.041 | 0.68 (0.55-0.84) | 0.015 | 0.65 (0.52-0.82) | 0.012 |
| S(5,-5)SumAverg | 0.16 (1.02) | -0.17 (0.96) | 0.71 (0.58-0.87) | 0.041 | 0.68 (0.55-0.84) | 0.015 | 0.66 (0.53-0.83) | 0.012 |
| S(2,-2)SumAverg | 0.16 (1.00) | -0.17 (0.98) | 0.71 (0.58-0.88) | 0.048 | 0.68 (0.55-0.84) | 0.017 | 0.64 (0.51-0.81) | 0.012 |
| S(3,0)SumAverg | 0.14 (1.00) | -0.16 (0.99) | 0.73 (0.60-0.90) | 0.057 | 0.70 (0.57-0.86) | 0.022 | 0.66 (0.53-0.83) | 0.012 |
| S(2,0)SumAverg | 0.14 (0.97) | -0.15 (1.02) | 0.74 (0.61-0.91) | 0.060 | 0.71 (0.58-0.88) | 0.035 | 0.66 (0.53-0.82) | 0.012 |
| S(1,-1)SumAverg | 0.13 (0.95) | -0.15 (1.04) | 0.75 (0.61-0.92) | 0.060 | 0.71 (0.57-0.88) | 0.039 | 0.65 (0.52-0.81) | 0.012 |
| S(4,0)SumAverg | 0.15 (1.01) | -0.16 (0.97) | 0.73 (0.59-0.89) | 0.056 | 0.70 (0.56-0.86) | 0.020 | 0.66 (0.53-0.83) | 0.013 |
| S(5,0)SumAverg | 0.15 (1.02) | -0.16 (0.97) | 0.73 (0.59-0.89) | 0.056 | 0.70 (0.57-0.86) | 0.020 | 0.67 (0.54-0.84) | 0.014 |
| WavEnLL_s-7 | -0.15 (1.04) | 0.17 (0.94) | 1.39 (1.13-1.70) | 0.049 | 1.45 (1.17-1.78) | 0.020 | 1.49 (1.19-1.87) | 0.015 |
| S(1,0)SumAverg | 0.11 (0.92) | -0.13 (1.08) | 0.78 (0.64-0.96) | 0.064 | 0.75 (0.61-0.93) | 0.083 | 0.67 (0.54-0.84) | 0.015 |
| WavEnLL_s-5 | -0.18 (1.05) | 0.19 (0.92) | 1.47 (1.19-1.80) | 0.035 | 1.51 (1.22-1.87) | 0.015 | 1.47 (1.17-1.85) | 0.023 |
| WavEnLL_s-4 | -0.18 (1.05) | 0.19 (0.92) | 1.46 (1.19-1.80) | 0.035 | 1.50 (1.21-1.85) | 0.015 | 1.43 (1.14-1.79) | 0.047 |

^*^ Only features with FDR-corrected P values less than 0.05 in Model 3 were present.

^a^ Crude model.

^b^ Estimates were adjusted for age and menopausal status.

^c^ Estimates were adjusted for age, menopausal status, molecular subtype, tumor stage, and histologic grade.

Abbreviations: SD, standard deviation; OR, odds ratio; CI, confidence interval.
