# Supplementary material for: Mammography radiomics features at diagnosis and progression-free survival among patients with breast cancer
Source: Br J Cancer. 2022 Sep 1;127(10):1886–92. doi: 10.1038/s41416-022-01958-5 (PMC9643418; doi:10.1038/s41416-022-01958-5)
Supplement: Supplementary file 6 — Supplementary Table S6 [file 41416_2022_1958_MOESM6_ESM.docx]

**Supplementary Table S6.** Mammography radiomics features at diagnosis associated with invasive disease-free survival among patients with breast cancer*, before multiple testing correlation.

| **Features** | **OR (95%CI)** | **Empirical P** |
| --- | --- | --- |
| CC_S(2,-2)SumAverg | 0.64 (0.51-0.80) | 1.12E-04 |
| CC_S(1,-1)SumAverg | 0.64 (0.42-0.87) | 1.40E-04 |
| CC_S(3,-3)SumAverg | 0.65 (0.52-0.81) | 1.45E-04 |
| CC_S(4,-4)SumAverg | 0.65 (0.52-0.82) | 1.85E-04 |
| CC_WavEnLL_s-6 | 1.53 (1.22-1.92) | 2.03E-04 |
| CC_S(5,-5)SumAverg | 0.66 (0.52-0.82) | 2.22E-04 |
| CC_S(2,0)SumAverg | 0.66 (0.53-0.82) | 2.31E-04 |
| CC_S(3,0)SumAverg | 0.66 (0.53-0.83) | 2.58E-04 |
| CC_S(4,0)SumAverg | 0.67 (0.53-0.83) | 3.22E-04 |
| CC_S(5,0)SumAverg | 0.67 (0.54-0.84) | 4.07E-04 |
| CC_S(1,0)SumAverg | 0.67 (0.54-0.84) | 4.33E-04 |
| CC_WavEnLL_s-7 | 1.48 (1.18-1.85) | 0.001 |
| CC_WavEnLL_s-5 | 1.48 (1.18-1.85) | 0.001 |
| CC_WavEnHL_s-5 | 0.69 (0.55-0.86) | 0.001 |
| CC_WavEnLL_s-4 | 1.44 (1.15-1.80) | 0.001 |
| CC_S(0,1)SumAverg | 0.71 (0.57-0.89) | 0.003 |
| CC_S(0,2)SumAverg | 0.72 (0.57-0.89) | 0.003 |
| CC_WavEnHL_s-6 | 0.73 (0.59-0.91) | 0.005 |
| CC_S(0,3)SumAverg | 0.73 (0.58-0.91) | 0.005 |
| CC_S(1,1)SumAverg | 0.73 (0.59-0.91) | 0.005 |
| CC_WavEnLH_s-6 | 0.73 (0.58-0.91) | 0.006 |
| CC_Perc.01. | 0.73 (0.58-0.92) | 0.006 |
| CC_S(0,4)SumAverg | 0.73 (0.59-0.92) | 0.006 |
| CC_WavEnLL_s-3 | 1.36 (1.09-1.69) | 0.007 |
| CC_S(0,5)SumAverg | 0.74 (0.59-0.92) | 0.008 |
| CC_Perc.10. | 0.74 (0.59-0.92) | 0.008 |
| CC_S(2,2)SumAverg | 0.75 (0.60-0.93) | 0.009 |
| CC_Mean | 0.74 (0.58-0.93) | 0.010 |
| CC_Perc.50. | 0.74 (0.58-0.93) | 0.010 |
| CC__MinNorm | 0.76 (0.61-0.94) | 0.014 |
| CC_S(3,3)SumAverg | 0.76 (0.61-0.95) | 0.014 |
| CC_S(4,4)SumAverg | 0.77 (0.62-0.96) | 0.020 |
| CC_WavEnLL_s-8 | 1.30 (1.04-1.62) | 0.020 |
| CC_Perc.90. | 0.76 (0.60-0.96) | 0.021 |
| CC_WavEnHL_s-7 | 0.77 (0.61-0.96) | 0.022 |
| CC_S(5,5)SumAverg | 0.78 (0.63-0.97) | 0.023 |
| CC_GrKurtosis | 1.29 (1.01-1.65) | 0.045 |
| MLO_GrKurtosis | 1.33 (1.03-1.70) | 0.026 |
| MLO_Skewness | 1.27 (1.02-1.58) | 0.031 |
| MLO_S(1,0)SumAverg | 0.80 (0.64-1.00) | 0.047 |

* Only features with empirical P values less than 0.05 in Model 3 were present.

Abbreviations: CC, cranio-caudal; MLO, mediolateral oblique.
