# Supplementary material for: Mammography radiomics features at diagnosis and progression-free survival among patients with breast cancer
Source: Br J Cancer. 2022 Sep 1;127(10):1886–92. doi: 10.1038/s41416-022-01958-5 (PMC9643418; doi:10.1038/s41416-022-01958-5)
Supplement: Supplementary file 7 — Supplementary Table S7 [file 41416_2022_1958_MOESM7_ESM.docx]

**Supplementary Table S7**. Associations of top independent mammography radiomics features at diagnosis with invasive disease-free survival among patients with breast cancer^*^, stratified by menopausal status and tumor characteristics.

|  | **S(1,-1)SumAverg** | | | **WavEnLL_s-6** | | |
| --- | --- | --- | --- | --- | --- | --- |
|  | **Controls,**  **mean (SD)** | **Cases,**  **mean (SD)** | **OR (95% CI)^a^** | **Controls,**  **mean (SD)** | **Cases,**  **mean (SD)** | **OR (95% CI)** |
| **By menopausal status** | | | | | | |
| No | 0.06 (0.98) | -0.36 (1.03) | 0.55 (0.42-0.74) | -0.15 (1.03) | 0.39 (0.88) | 1.94 (1.44-2.61) |
| Yes | 0.29 (0.87) | 0.16 (0.97) | 0.86 (0.59-1.26) | -0.28 (1.08) | -0.08 (0.90) | 1.05 (0.73-1.49) |
| P for interaction |  |  | 0.034 |  |  | 0.010 |
| **By molecular subtype** | | | | | | |
| Luminal A | 0.57 (0.63) | 0 (1.39) | 0.40 (0.16-1.01) | -0.58 (0.93) | 0.27 (1.30) | 2.34 (1.01-5.39) |
| Luminal B | 0.09 (1.02) | 0 (0.94) | 0.81 (0.60-1.08) | -0.21 (1.07) | 0.13 (0.88) | 1.48 (1.10-1.99) |
| HER2 positive | -0.06 (0.92) | -0.38 (0.96) | 0.68 (0.37-1.25) | 0.09 (0.99) | 0.19 (0.97) | 0.93 (0.53-1.63) |
| TNBC | 0.13 (0.72) | -0.30 (1.11) | 0.52 (0.28-0.96) | -0.23 (1.08) | 0.41 (0.82) | 2.14 (1.09-4.18) |
| Indeterminate | 0.50 (0.92) | -0.50 (1.29) | 0.24 (0.09-0.64) | -0.15 (0.95) | 0.17 (0.99) | 2.00 (0.86-4.70) |
| P for interaction |  |  | 0.014 |  |  | 0.259 |
| **By tumor stage** | | | | | | |
| Ⅰ | 0.08 (1.03) | -0.51 (1.35) | 0.58 (0.37-0.91) | -0.32 (1.20) | 0.14 (1.30) | 1.50 (0.96-2.32) |
| Ⅱ | 0.09 (0.86) | -0.14 (0.94) | 0.65 (0.47-0.91) | -0.15 (0.97) | 0.22 (0.83) | 1.70 (1.23-2.35) |
| Ⅲ | 0.40 (1.10) | -0.03 (1.03) | 0.69 (0.47-1.03) | -0.09 (1.02) | 0.17 (0.90) | 1.28 (0.82-2.00) |
| P for interaction |  |  | 0.860 |  |  | 0.601 |
| **By histologic grade** | | | | | | |
| Ⅰ-Ⅱ | 0.15 (1.05) | -0.19 (1.13) | 0.60 (0.43-0.84) | -0.20 (1.16) | 0.08 (0.97) | 1.44 (1.05-1.98) |
| Ⅲ | 0.12 (0.86) | -0.11 (0.99) | 0.68 (0.51-0.92) | -0.18 (0.95) | 0.25 (0.88) | 1.62 (1.19-2.21) |
| P for interaction |  |  | 0.299 |  |  | 0.597 |

^*^ According to the results of Logistic models in Table 2 and correlation matrix analysis, S(1,-1)SumAverg and WavEnLL_s-6 were identified as the top independent mammography radiomics features at diagnosis of invasive disease-free survival among patients with breast cancer in cluster 1 and cluster 2.

^a^ ORs were adjusted for age, menopausal status, molecular subtype, tumor stage, histologic grade.

Abbreviations: SD, standard deviation; OR, odds ratio; CI, confidence interval; HER2, human epidermal growth factor receptor 2; TNBC, triple-negative breast cancer.
