# Supplementary material for: Mammography radiomics features at diagnosis and progression-free survival among patients with breast cancer
Source: Br J Cancer. 2022 Sep 1;127(10):1886–92. doi: 10.1038/s41416-022-01958-5 (PMC9643418; doi:10.1038/s41416-022-01958-5)
Supplement: Supplementary file 8 — Supplementary Table S8 [file 41416_2022_1958_MOESM8_ESM.docx]

**Supplementary Table S8.** Associations between top features and tumor characteristics among patients with breast cancer in the linear models^a^.

|  |  | **S(1,-1)SumAverg** | | **WavEnLL_s-6** | |
| --- | --- | --- | --- | --- | --- |
|  |  | **OR (95% CI)** | **P** | **OR (95% CI)** | **P** |
| Tumor stage | Ⅰ | Reference |  | Reference |  |
|  | Ⅱ | 1.04 (0.81-1.34) | 0.763 | 1.26 (0.97-1.62) | 0.079 |
|  | Ⅲ | 1.14 (0.86-1.51) | 0.350 | 1.37 (1.03-1.81) | 0.029 |
| Molecular subtype | Luminal A | Reference |  | Reference |  |
|  | Luminal B | 0.88 (0.58-1.34) | 0.553 | 1.03 (0.68-1.58) | 0.874 |
|  | HER2 positive | 0.64 (0.40-1.02) | 0.062 | 1.29 (0.80-2.09) | 0.293 |
|  | TNBC | 0.78 (0.49-1.24) | 0.292 | 1.17 (0.73-1.88) | 0.512 |
|  | Indeterminate | 0.77 (0.45-1.30) | 0.323 | 1.17 (0.69-2.00) | 0.565 |
| Histologic grade | Ⅰ-Ⅱ | Reference |  | Reference |  |
|  | Ⅲ | 1.01 (0.83-1.22) | 0.956 | 1.13 (0.93-1.38) | 0.205 |

^a^ Estimates were adjusted for age and menopausal status.

Abbreviations: OR, odds ratio; CI, confidence interval; HER2, human epidermal growth factor receptor 2; TNBC, triple-negative breast cancer.
