# Supplementary material for: Mammography radiomics features at diagnosis and progression-free survival among patients with breast cancer
Source: Br J Cancer. 2022 Sep 1;127(10):1886–92. doi: 10.1038/s41416-022-01958-5 (PMC9643418; doi:10.1038/s41416-022-01958-5)
Supplement: Supplementary file 9 — Supplementary Table S9 [file 41416_2022_1958_MOESM9_ESM.docx]

**Supplementary Table S9**. Genes associated with top independent mammography radiomics features at diagnosis among patients with breast cancer in the linear models^a^.

|  |  | **Tumor tissue** | | **Adjacent normal tissue** | |
| --- | --- | --- | --- | --- | --- |
|  | **Gene symbol** | **OR (95%CI)** | **P*** | **OR (95%CI)** | **P*** |
| **S(1,-1)SumAverg** | MOV10L1 | 1.66 (1.39-1.98) | 0.005 | 1.17 (0.90-1.52) | 0.916 |
|  | LRP1B | 1.61 (1.35-1.93) | 0.011 | 1.17 (0.94-1.46) | 0.916 |
|  | ZFY | 1.55 (1.29-1.86) | 0.014 | 0.82 (0.66-1.03) | 0.912 |
|  | PITX2 | 1.58 (1.32-1.89) | 0.014 | 1.13 (0.91-1.40) | 0.916 |
|  | TRAPPC12 | 1.56 (1.30-1.87) | 0.014 | 1.06 (0.84-1.34) | 0.972 |
|  | MMGT1 | 0.62 (0.50-0.76) | 0.018 | 0.89 (0.70-1.13) | 0.922 |
|  | PPP2R2C | 0.67 (0.55-0.81) | 0.047 | 1.02 (0.80-1.29) | 0.991 |
| **WavEnLL_s-6** | CSNK2A1 | 1.80 (1.46-2.21) | 0.005 | 0.83 (0.67-1.03) | 0.797 |

^a^ Estimates were adjusted for age, menopausal status, molecular subtype, tumor stage, and histologic grade.

* FDR-corrected P value.

Abbreviations: OR, odds ratio; CI, confidence interval.
