# Supplementary material for: Mammography radiomics features at diagnosis and progression-free survival among patients with breast cancer
Source: Br J Cancer. 2022 Sep 1;127(10):1886–92. doi: 10.1038/s41416-022-01958-5 (PMC9643418; doi:10.1038/s41416-022-01958-5)
Supplement: Supplementary file 10 — Supplementary Table S10 [file 41416_2022_1958_MOESM10_ESM.docx]

**Supplementary Table S10.** Clinical characteristics of breast cancer patients with mammography and without mammography.

|  |  | Without mammography | With mammography | P |
| --- | --- | --- | --- | --- |
| Number |  | 2718 | 3737 |  |
|  |  | mean (SD) | mean (SD) |  |
| Age, years |  | 49.45 (10.40) | 47.97 (9.93) | <0.001 |
|  |  | N (%) | N (%) |  |
| Menopausal status | No | 1538 (56.6) | 2375 (63.6) | <0.001 |
|  | Yes | 1180 (43.4) | 1362 (36.4) |  |
| Molecular subtype | Luminal A | 261 (9.6) | 394 (10.5) | 0.003 |
|  | Luminal B | 1472 (54.2) | 2156 (57.7) |  |
|  | HER2 positive | 344 (12.7) | 386 (10.3) |  |
|  | TNBC | 320 (11.8) | 415 (11.1) |  |
|  | Indeterminate | 321 (11.8) | 386 (10.3) |  |
| Tumor stage | Tis | 88 (3.2) | 158 (4.2) | <0.001 |
|  | Ⅰ | 437 (16.1) | 857 (22.9) |  |
|  | Ⅱ | 1038 (38.2) | 1706 (45.7) |  |
|  | Ⅲ | 730 (26.9) | 738 (19.7) |  |
|  | Ⅳ | 150 (5.5) | 83 (2.2) |  |
|  | Unknown | 275 (10.1) | 195 (5.2) |  |
| Histologic grade | Ⅰ-Ⅱ | 871 (32.0) | 1407 (37.7) | <0.001 |
|  | Ⅲ | 1131 (41.6) | 1648 (44.1) |  |
|  | Unknown | 716 (26.3) | 682 (18.2) |  |
| Hormone therapy | No | 942 (34.7) | 1058 (28.3) | <0.001 |
|  | Yes | 1776 (65.3) | 2679 (71.7) |  |
| Chemotherapy | No | 418 (15.4) | 412 (11.0) | <0.001 |
|  | Yes | 2300 (84.6) | 3325 (89.0) |  |
| Radiotherapy | No | 1946 (71.6) | 2693 (72.1) | 0.701 |
|  | Yes | 772 (28.4) | 1044 (27.9) |  |

Abbreviations: HER2, human epidermal growth factor receptor 2; TNBC, triple-negative breast cancer.
