# Supplementary material for: Mammography radiomics features at diagnosis and progression-free survival among patients with breast cancer
Source: Br J Cancer. 2022 Sep 1;127(10):1886–92. doi: 10.1038/s41416-022-01958-5 (PMC9643418; doi:10.1038/s41416-022-01958-5)
Supplement: Supplementary file 11 — Supplementary Table S11 [file 41416_2022_1958_MOESM11_ESM.docx]

**Supplementary Table S11.** Clinical characteristics of breast cancer patients whose tumors have been sequenced for RNA or not.

|  |  | **Not sequenced** | **Sequenced** | **P** |
| --- | --- | --- | --- | --- |
| Number |  | 322 | 96 |  |
|  |  | mean (SD) | mean (SD) |  |
| Age, years |  | 47.98 (9.51) | 50.30 (10.21) | 0.040 |
|  |  | N (%) | N (%) |  |
| Menopausal status | No | 211 (65.5) | 48 (50.0) | 0.009 |
|  | Yes | 111 (34.5) | 48 (50.0) |  |
| Molecular subtype | Luminal A | 19 (5.9) | 6 (6.2) | 0.748 |
|  | Luminal B | 186 (57.8) | 52 (54.2) |  |
|  | HER2 positive | 43 (13.4) | 17 (17.7) |  |
|  | TNBC | 49 (15.2) | 16 (16.7) |  |
|  | Indeterminate | 25 (7.8) | 5 (5.2) |  |
| Tumor stage | Ⅰ | 67 (20.8) | 15 (15.6) | 0.487 |
|  | Ⅱ | 167 (51.9) | 51 (53.1) |  |
|  | Ⅲ | 88 (27.3) | 30 (31.2) |  |
| Histologic grade | Ⅰ-Ⅱ | 130 (40.4) | 36 (37.5) | 0.699 |
|  | Ⅲ | 192 (59.6) | 60 (62.5) |  |
| Hormone therapy | No | 15 (4.7) | 2 (2.1) | 0.408 |
|  | Yes | 307 (95.3) | 94 (97.9) |  |
| Chemotherapy | No | 113 (35.1) | 43 (44.8) | 0.109 |
|  | Yes | 209 (64.9) | 53 (55.2) |  |
| Radiotherapy | No | 209 (64.9) | 59 (61.5) | 0.619 |
|  | Yes | 113 (35.1) | 37 (38.5) |  |

Abbreviations: HER2, human epidermal growth factor receptor 2; TNBC, triple-negative breast cancer.
