# Supplementary material for: Mammography radiomics features at diagnosis and progression-free survival among patients with breast cancer
Source: Br J Cancer. 2022 Sep 1;127(10):1886–92. doi: 10.1038/s41416-022-01958-5 (PMC9643418; doi:10.1038/s41416-022-01958-5)
Supplement: Supplementary file 13 — Figure legends of Supplementary Figure S1 [file 41416_2022_1958_MOESM13_ESM.docx]

**Figure legends of Supplementary Figure S1**

**Supplementary Figure S1.** Correlations among lead mammography radiomics features at diagnosis.
